# Lack of association between glucocorticoid use and presence of the metabolic syndrome in patients with rheumatoid arthritis: a cross-sectional study

**DOI:** 10.1186/ar2578

**Published:** 2008-12-17

**Authors:** Tracey E Toms, Vasileios F Panoulas, Karen MJ Douglas, Helen R Griffiths, George D Kitas

**Affiliations:** 1 Department of Rheumatology, Dudley Group of Hospitals NHS Trust, Russells Hall Hospital, Dudley, West Midlands, DY1 2HQ, UK; 2 Arthritis Research Campaign Epidemiology Unit, Manchester University, Oxford Road, Manchester, M13 9PL, UK; 3 Life and Health Sciences, Aston University, Aston Triangle, Birmingham, West Midlands, B4 7ET, UK

## Abstract

**Introduction:**

Rheumatoid arthritis (RA) associates with excessive cardiovascular morbidity and mortality, attributed to both traditional and novel cardiovascular risk factors. The metabolic syndrome, a cluster of classical cardiovascular risk factors, including hypertension, obesity, glucose intolerance, and dyslipidaemia, is highly prevalent in RA. Reports suggest that long-term glucocorticoid (GC) use may exacerbate individual cardiovascular risk factors, but there have been no studies in RA to assess whether it associates with the metabolic syndrome. We examined whether GC exposure associates with the presence of metabolic syndrome in patients with RA.

**Methods:**

RA patients (n = 398) with detailed clinical and laboratory assessments were categorised into three groups according to GC exposure: no/limited (<3 months) exposure (NE), low-dose (<7.5 mg/day) long-term exposure (LE), and medium-dose (greater than or equal to 7.5 mg to 30 mg/day) long-term exposure (ME). The metabolic syndrome was defined using the National Cholesterol Education Programme III guidelines. The association of GC exposure with the metabolic syndrome was evaluated using binary logistic regression.

**Results:**

The metabolic syndrome was present in 40.1% of this population and its prevalence did not differ significantly between the GC exposure groups (NE 37.9% versus LE 40.7% versus ME 50%, *P *= 0.241). Binary logistic regression did not demonstrate any increased odds for the metabolic syndrome when comparing ME with LE (odds ratio = 1.64, 95% confidence interval 0.92 to 2.92, *P *= 0.094) and remained non significant after adjusting for multiple potential confounders.

**Conclusions:**

Long-term GC exposure does not appear to associate with a higher prevalence of the metabolic syndrome in patients with RA. The components of the metabolic syndrome may already be extensively modified by other processes in RA (including chronic inflammation and treatments other than GCs), leaving little scope for additive effects of GCs.

## Introduction

An association between rheumatoid arthritis (RA) and increased cardiovascular disease burden has been recognised for many years [1]. This results in excess mortality and reduced lifespan compared with the general population [2,3]. Many factors may contribute to the increased cardiovascular risk, including classical risk factors such as smoking, diabetes, hypertension, and obesity and novel risk factors such as systemic inflammation, a prothrombotic state, and hyperhomocystinaemia [4].

Glucocorticoids (GCs) are known to have beneficial effects in controlling rheumatoid inflammation [5,6] but their use has been curbed due to their adverse effects. They are used increasingly as a short-term measure to induce rapid reduction in disease activity whilst awaiting the effects of slower acting disease-modifying anti-rheumatic drugs (DMARDs) [7]. Long-term use of GCs is controversial due to an undesirable side effect profile that includes dyslipidaemia [8], hyperglycaemia [9], insulin resistance [10], hypertension [11,12], and central obesity [11]: these may collectively contribute to the development of the metabolic syndrome [13,14] and atherosclerosis [15,16].

The metabolic syndrome, first described by Reaven in 1988 [17], is a cluster of classical cardiovascular risk factors (obesity, glucose intolerance, dyslipidaemia, and hypertension) thought to associate with cardiovascular risk beyond the sum of its individual components [18], although this has recently been questioned [19]. It is estimated that the metabolic syndrome affects 20% to 30% of the adult population in developed countries [20-22] but its prevalence varies according to the definition used as well as environmental and genetic differences between populations [22]. As a consequence of physical inactivity and poor diet, rates of obesity and diabetes are increasing worldwide [23,24], with an expected exponential rise in the percentage of the population affected by the metabolic syndrome [23]. The pathogenesis underlying the clustering of cardiovascular risk factors remains unclear: genetic predisposition, obesity, and inflammation have been suggested to be involved [25,26]. A recent study has shown that patients with RA have a higher prevalence of metabolic syndrome than controls [27], although another study has demonstrated an equally high prevalence between Mediterranean RA patients and local general population controls [28]. In the present cross-sectional study, we addressed the hypothesis that RA patients receiving long-term GC will have an increased prevalence of each of the individual components of the metabolic syndrome (dyslipidaemia, hypertension, hyperglycaemia, and central obesity) as well as the metabolic syndrome itself.

## Materials and methods

RA patients (n = 398) fulfilling the revised American College of Rheumatology criteria [29] were recruited from routine outpatient clinics at the Department of Rheumatology, Dudley Group of Hospitals NHS Trust (Dudley, West Midlands, UK). Characteristics of the study population are shown in Table 1. Local ethics committee approval was granted for the study and all participants provided informed consent.

**Table 1:** Demographics and clinical and laboratory characteristics of the glucocorticoid exposure groups

	NE (n = 281)	LE (n = 58)	ME (n = 59)	*P *value
General demographics				
Age, years	62 (53.03–68.56)	66.01 (59–71.47)	66.6 (60.7–71.6)	0.002
Gender female, number (percentage)	214 (76.2)	41 (70.7)	36 (61)	0.053
RA characteristics				
RF-positive, number (percentage)	210 (76.4)	42 (73.7)	43 (75.4)	0.909
Anti-CCP-positive, number (percentage)	181 (67.5)	36 (63.2)	41 (73.2)	0.517
Disease duration, years	7 (3–15)	16 (10–25.5)	18 (10–39)	<0.001
Disease activity				
CRP, mg/L	8 (5–18)	8 (5–17)	9 (5–28)	0.421
ESR, mm/h	20 (10–38)	15.5 (6.75–29.25)	26 (12–45)	0.031
DAS-28	4.15 ± 1.38	4.09 ± 1.37	4.59 ± 1.47	0.080
Disease severity				
HAQ	1.38 (0.5–2)	1.63 (0.72–2.16)	2.13 (1.63–2.5)	<0.001
Extra-articular disease, number (percentage)	180 (64.1)	42 (72.4)	45 (76.3)	0.124
Joint replacement surgery, number (percentage)	65 (23.1)	21 (36.2)	29 (49.2)	<0.001
Medications				
Methotrexate, number (percentage)	161 (57.3)	35 (60.3)	28 (47.5)	0.305
Sulphasalazine, number (percentage)	96 (34.2)	11 (19)	11 (18.6)	0.009
Hydroxychloroquine, number (percentage)	57 (20.3)	13 (22.4)	10 (16.9)	0.754
Leflunomide, number (percentage)	11 (3.9)	1 (1.7)	4 (6.8)	0.374
Anti-TNF, number (percentage)	29 (10.3)	9 (15.5)	7 (11.9)	0.518
NSAIDS/COX II, number (percentage)	82 (29.2)	15 (25.9)	13 (22)	0.508
Risk factors for the MetS				
Waist, cm	97.12 ± 12.39	97.35 ± 14.3	100.9 ± 15.19	0.172
Trigylcerides, mmol/L	1.2 (0.9–1.6)	1.2 (0.9–1.63)	1.4 (1.1–1.8)	0.010
Systolic blood pressure, mm Hg	140 (126.25–150)	142 (125.75–159)	145 (135–160)	0.022
Diastolic blood pressure, mm Hg	78.66 ± 10.85	79.97 ± 10.82	79.06 ± 13.06	0.716
HDL, mmol/L	1.5 (1.2–1.8)	1.7 (1.38–2.03)	1.7 (1.4–2)	0.011
LDL, mmol/L	5.2 ± 1.07	5.31 ± 1.27	5.25 ± 1.54	0.798
Criteria for MetS				
Waist M, number (percentage)	163 (65.2)	32 (65.3)	36 (69.2)	0.853
Triglycerides M, number (percentage)	96 (34.2)	24 (41.4)	31 (52.5)	0.026
Hypertension M, number (percentage)	220 (78.3)	45 (77.6)	55 (93.2)	0.027
HDL M, number (percentage)	94 (33.5)	20 (34.5)	24 (40.7)	0.570
Fasting plasma glucose M, number (percentage)	38 (13.5)	9 (15.8)	14 (24.1)	0.125
Patients fulfilling criteria of MetS				
NCEP, number (percentage)	105 (37.9)	22 (40.7)	28 (50.0)	0.241

Results expressed as number (percentage), median (25th–75th percentile), or mean ± standard deviation, as appropriate. Anti-CCP, anti-cyclic citrullinated peptide; anti-TNF, anti-tumour necrosis factor therapy; CRP, C-reactive protein; HAQ, health assessment questionnaire; DAS-28, 28-joint disease activity score; ESR, erythrocyte sedimentation rate; Triglycerides M, triglycerides of at least 1.7 mmol/L or on drug treatment for elevated triglycerides; Waist M, waist circumference of greater than 102 cm in males and greater than 88 cm in females. Fasting plasma glucose M, fasting plasma glucose of at least 6.1 mmol/L or on drug treatment for elevated blood glucose; HDL, high-density lipoprotein; HDL M, high-density lipoprotein level of less than 1.0 mmol/L in males or less than 1.3 mmol/L in females or on drug treatment for low HDL; Hypertension M, systolic blood pressure of at least 130/85 mm Hg or on antihypertensive medication; LDL, low-density lipoprotein; LE, low-dose/long-term exposure; ME, moderate-dose/long-term exposure; MetS, metabolic syndrome; NCEP, National Cholesterol Education Programme; NE, none/limited exposure; NSAIDs/COX II, nonsteroidal anti-inflammatory drugs/cyclooxygenase II inhibitors; RA, rheumatoid arthritis; RF, rheumatoid factor;

All patients underwent the same baseline evaluation prospectively. The following details were recorded: basic demographics (that is, age, gender, height, weight, and waist circumference), a full medical history (including specific details regarding RA and cardiovascular disease), and current disease activity and physical function, using the 28-joint disease activity score (DAS-28) [30] and health assessment questionnaire (HAQ) [31], respectively. All current medications, including anti-rheumatic drugs, analgesics, and cardiovascular drugs such as statins or anti-hypertensives, were documented. Information regarding GC (oral prednisolone) dose and duration of exposure was recorded and categorised into none or low-dose (<7.5 mg of prednisolone daily), limited duration (<3 months) GC exposure (no exposure, NE), low-dose long-term (>6 months) (low exposure, LE), or medium-dose (prednisolone at least 7.5 to 30 mg daily for more than 6 months) long-term (medium exposure). There were no patients exposed to high-dose long-term GC. Baseline blood samples, including fasting lipid profiles (total cholesterol, triglycerides (TGs), and high-density lipoproteins (HDLs)), erythrocyte sedimentation rate (ESR), C-reactive protein, fasting glucose, insulin, and rheumatoid factor, were taken. All blood tests were analysed by a single laboratory. The metabolic syndrome was classified by National Cholesterol Education Programme III (NCEP III) criteria [32]: fasting plasma glucose of at least 6.1 mmol/L or on drug treatment for elevated blood glucose, systolic blood pressure of at least 130/85 mm Hg or on drug treatment for elevated blood pressure, TG at least 1.7 mmol/L or drug treatment for elevated TG, HDL in males of less than 1.0 mmol/L and in females of less than 1.3 mmol/L or on drug treatment for a low HDL, and waist circumferences in males of greater than 102 cm and in females of greater than 88 cm, with the metabolic syndrome defined as the presence of at least three of these criteria [32].

Analysis of the data was carried out using SPSS 14.0 (SPSS Inc., Chicago, IL, USA). The Kolmogorov-Smirnov test was used to evaluate the distribution of each parameter. Values were expressed as mean ± standard deviation, median (25th–75th percentile), or number (percentage), as appropriate. Comparisons were performed by analysis of variance, Kruskal-Wallis, and chi-square test for normally distributed, non-normally distributed, and categorical variables, respectively. To evaluate the independence of the association between GC exposure and the metabolic syndrome, binary logistic regression analysis was used.

## Results

### Descriptive characteristics of the population studied

Median (interquartile range) age in the total cohort was 63.08 (55.46 to 69.62) years, disease duration was 10 (4 to 18) years, and 292 (73%) of the patients were female. Three hundred forty patients (87.4%) were receiving DMARDs (two thirds as monotherapy and the remaining as combination therapy), of whom 225 (56.3%) were taking methotrexate, 118 (29.5%) sulphasalazine, 80 (20%) hydroxycholoroquine, 16 (4%) leflunomide, and 46 (11.5%) anti-tumour necrosis factor (anti-TNF) biologics; 111 patients (27.8%) were on nonsteroidal anti-inflammatory drugs or cyclooxygenase II inhibitors. One third of the cohort, 117 patients (29.4%), were taking GCs, with similar numbers in the LE and ME groups (58 versus 59 patients, respectively). Other drugs included statins in 83 (20.8%) and antihypertensives in 177 (44.5%) patients. Further baseline characteristics of the study population are described in detail in previous papers by our group [12,33].

### Univariate analysis

Patients in the LE and ME groups were older (*P *= 0.002) and had longer disease duration (*P *< 0.001) and higher ESR (*P *= 0.031) and HAQ (*P *< 0.001) and more frequent joint replacement surgery (*P *< 0.001) than those in the NE group (Table 1). There were no significant differences between LE and ME. Of the risk factors for the metabolic syndrome, TG, systolic blood pressure, and HDL were all higher in patients receiving long-term GCs (LE or ME). TGs were significantly higher in the ME group (*P *= 0.010) compared with NE, but no significant difference was noted between LE and NE. Systolic blood pressure increased as GC exposure increased (NE 140 (126.25 to 150) mm Hg versus LE 142 (125.75 to 159) mm Hg versus ME 145 (135 to 160) mm Hg, *P *= 0.022). Interestingly, HDL levels were higher in the LE and ME groups (*P *= 0.011).

The individual components that comprise the metabolic syndrome were analysed to assess the proportion of patients in each of the GC exposure groups fulfilling the NCEP III-defined cut off levels. TG levels adequate for contributing as a component of the metabolic syndrome were observed in a significantly higher proportion of patients in the LE and ME groups compared with NE (*P *= 0.026). The NCEP III cut off for hypertension was present in a significantly higher proportion of patients in the ME group (*P *= 0.027) but not in the LE group. The other individual components that contribute to the definition of the metabolic syndrome (waist circumference, HDL, and fasting plasma glucose) were observed in similar proportions of patients within the NE, LE, and ME groups.

On combining the individual components to fulfil the NCEP III criteria for the metabolic syndrome (presence of at least three of the components), there was no evidence of a significant association between the degree of GC exposure and the presence of the metabolic syndrome (NE 105 (37.9%) versus LE 22 (40.7%) versus ME 28 (50%), *P *= 0.241). Factors identified to be significantly associated with both the metabolic syndrome and GC exposure, thus acting as potential confounders, included age (*P *= 0.001 and *P *= 0.002, respectively), RA disease duration (*P *= 0.008 and *P *< 0.001), ESR (*P *= 0.006 and *P *= 0.031), and HAQ (*P *= 0.036 and *P *< 0.001).

### Multivariate analysis

The association between GC exposure and the frequency of the metabolic syndrome was assessed using a logistic regression model (Table 2). The analysis did not demonstrate a statistically significant association between LE (odds ratio (OR) = 1.13 (0.62 to 2.04), *P *= 0.695) or ME (OR = 1.64 (0.92 to 2.92), *P *= 0.094) and the metabolic syndrome in either the crude model or following adjustment for all of the potential confounders mentioned above (age, ESR, disease duration, and HAQ; for completeness, gender was also added to the model): LE OR = 1.15 (0.61 to 2.19) (*P *= 0.670) and ME OR = 1.4 (0.72 to 2.58) (*P *= 0.340). For reasons of colinearity, ESR and DAS could not be included in the same model. However, to ensure that disease activity *per se *(rather than ESR alone) did not alter the results, the model was repeated including DAS instead of ESR. Despite this, no significant association was found between LE (OR = 1.189, *P *= 0.601) or ME (OR = 1.453, *P *= 0.262) and the metabolic syndrome.

**Table 2:** Odds ratios for the metabolic syndrome (NCEP III) in comparison with the amount of steroid exposure

	LD/LTE		MD/LTE	
		
	OR (95% CI)	*P *value	OR (95% CI)	*P *value
Crude	1.13 (0.62–2.04)	0.695	1.64 (0.92–2.92)	0.094
Model a	0.998 (0.54–1.83)	0.994	1.37 (0.75–2.48)	0.304
Model b	1.15 (0.61–2.19)	0.670	1.4 (0.72–2.58)	0.340

Crude = uncorrected data. Model a = crude data plus adjustment for age and gender. Model b = model a plus adjustment for markers of disease activity/severity (disease duration, erythrocyte sedimentation rate levels, and health assessment questionnaire). CI, confidence interval; LD/LTE, low-dose/long-term exposure; MD/LTE, moderate-dose/long-term exposure; NCEP III, National Cholesterol Education Programme III; OR, odds ratio.

## Discussion

This cross-sectional observational study has demonstrated that long-term exposure to GCs, particularly at medium doses, is associated with increased prevalence of two of the components of the metabolic syndrome (high TG and hypertension), but not any of the other components (low HDL, obesity, and glucose intolerance) or the presence of the metabolic syndrome itself. We have presented data having used the NECP III criteria for metabolic syndrome as they are the most up-to-date and widely used, allowing comparison of our results with those of other studies in RA and other conditions. However, for the purpose of completeness, we have also analysed the data on the basis of other metabolic syndrome classification criteria, including those of the World Health Organisation [34], the International Diabetes Federation [35], and the European Group for the Study of Insulin Resistance [36], in order to ensure that the definition used would not affect the outcome of this study. The lack of any association between GC exposure and the presence of the metabolic syndrome in this RA cohort was present irrespective of the metabolic syndrome criteria used (data not shown).

Although no statistically significant association between GC exposure and the metabolic syndrome was found, the multivariate analysis did demonstrate a trend in the OR as steroid exposure increased (LE 1.13 (0.62 to 2.04) versus ME 1.64 (0.92 to 2.92)). Thus, a possible limitation of the present study may be that, despite the fact that it is by far the largest to date to assess metabolic syndrome in RA, its power may not have been sufficient to detect such an association, although the clinical significance of such a weak association would then come into question. The most important limitation of this study, however, is its cross-sectional design, which does not allow definitive interpretation of the causality and directionality of the associations found. Notwithstanding these limitations, this study has addressed an original question that has not been addressed elsewhere in the literature in a large, very well-characterised RA population, with prospective data collection, thus allowing adjustment for many potential confounders and minimising selection and recall bias or missing values, which are all common problems in retrospective studies.

The absence of a relationship between long-term GC use and the presence of the metabolic syndrome may be explained by physical and metabolic changes occurring as part of the disease process or as an indirect consequence of treatments other than steroids [37-39]. It is possible that the beneficial effects produced by suppressing the impact of inflammation on the metabolic syndrome may outweigh the harmful effects of GCs. For example, in RA, many components of the lipid profile are suppressed by the ongoing inflammatory burden, including HDL [40]. However, suppression of the inflammatory load via drug use (for example, DMARDs) produces a significant increase in lipid levels, particularly HDL [41]. Conversely, it may be that patients with RA have significantly modified their risk factors for the metabolic syndrome as a consequence of inflammation and that the addition of GC use cannot worsen these further.

Central obesity is associated with increased cardiovascular risk and is one of the components of the metabolic syndrome. GC use has been shown to redistribute body fat and result in central obesity with relative sparing of the extremities [42]. Long-term GC use can also be complicated by the development of a steroid-induced myopathy [37-39,43], which may further exacerbate the imbalance between fat mass and muscle bulk. Although steroid myopathy primarily manifests with proximal muscle weakness in the absence of muscle atrophy [44], muscle wasting may occur as a consequence of a reduction in functional capacity. The systemic inflammation associated with RA causes a hypercatabolic state, which, together with a sedentary lifestyle, results in a loss of muscle bulk and an increased tendency to gain fat in the presence of stable or even slightly decreased weight, often referred to as rheumatoid cachexia [45]. It has been reported that almost two thirds of patients with RA have features of rheumatoid cachexia [46], and a recent study has demonstrated the need for redefining the body mass index in RA to take into account these changes [33]. It is therefore possible that, in RA patients, alterations in body fat/muscle may have already occurred as part of the disease process and thus any additional changes that are induced by long-term GC exposure may be insignificant. Alternatively, better control of inflammation due to steroid usage may counterbalance some of the body composition abnormalities that would have otherwise occurred. The latter possibility would be supported by our findings in RA patients treated with anti-TNF biologics [47].

A relationship between RA and abnormalities in the lipid profile has been observed for several decades [48], with the key contributing factor thought to be inflammation [49]. The typical pattern of dyslipidaemia seen in active RA is a reduction in total cholesterol, low-density lipoprotein, and HDL [40]. Data regarding TG levels in RA are conflicting, with some studies reporting an increase [50] and others a decrease [51]. The metabolic syndrome definition takes in to account only two components of the lipid profile, TG and HDL. GC use, independent of dose, has been shown to increase HDL levels [52-54]. Endogenous GC hormones regulate HDL plasma concentrations by increasing synthesis and secretion by the liver [55]. Consequently, high doses of exogenous GCs such as those being received by RA patients are likely to accelerate this pathway. Therefore, in RA, HDL levels previously suppressed by disease activity will rise, producing a less atherogenic profile. This was reflected in our study, in which an increase in HDL levels was seen in patients receiving long-term GCs (*P *= 0.011). The observed rise in HDL levels may also be explained by the indirect effects of GCs. Patients requiring GCs as part of their disease management are likely to have aggressive disease, which contributes to a reduction in mobility as a result of joint stiffness and damage. Therefore, GC use in this subset of patients may result in suppression of disease activity and allow patients to lead a more active lifestyle with a further beneficial effect on HDL levels [56].

Elevation of TG levels in patients receiving GCs on a long-term basis has also been demonstrated [57,58]. However, many of the studies have been limited due to the lack of control for other potential lipid-influencing factors such as metabolic, nutritional, or comorbid conditions. Although the proportion of patients fulfilling the NCEP III-specified cut off levels for TGs was significantly higher in the group receiving long-term GCs in this study (*P *= 0.026), this alone was not sufficient to impact significantly on the overall prevalence of the metabolic syndrome, possibly being counterbalanced by increases in HDL, producing an overall less atherogenic lipid profile.

Shortly after the discovery of GC by Hench and colleagues [59] in 1948, it became apparent that patients were frequently developing adverse effects, including hypertension and diabetes [60]. Despite this, there is limited evidence to support these initial observations. In the general population, studies attempting to assess the association between GC use and hypertension have been hampered by poor study design [61], thus resulting in a distinct lack of direct evidence to support this relationship. In RA, this association has been studied in more detail, with two recent studies demonstrating an increase in blood pressure in patients treated with long-term medium-dose (≥ 7.5 mg) GC [12,62]. One of these studies, a large cross-sectional study, confirmed an association independent of other risk factors for hypertension and RA disease activity or severity [12]. Although it appears that there is a link between GC exposure and hypertension in RA, it has also been speculated that RA itself may predispose patients to an increased prevalence of hypertension [63]. The data produced in the present study show a significant increase in the numbers of patients fulfilling the NCEP III-specified cut off levels for hypertension (= 130/85 mm Hg or taking anti-hypertensives) in those receiving long-term GC. It may be that, despite hypertension being prevalent in RA, there is still scope for further modification through the actions of GCs. Irrespective of this, it appears again that the increased frequency of patients satisfying both the hypertensive and hypertrigylceridaemic components of the metabolic syndrome in the steroid-treated group is not sufficient to also increase the frequency of those qualifying for a diagnosis of the metabolic syndrome.

GC-induced hyperglycaemia is a well-recognised complication of long-term GC use [64]. It is known that patients with active RA have impaired glucose handling and that glucose metabolism may be affected directly or indirectly by inflammatory mediators [65]. Several studies have demonstrated an association between GC use and decreased insulin sensitivity [10,66,67]. However, there is smaller bank of conflicting data demonstrating that GC use in RA paradoxically restores glucose handling to normal whilst also inducing hyperinsulinism [68]. In the present RA population, it is possible that GC use restored glucose handling to normal via anti-inflammatory mechanisms, hence explaining why this component was not significant in the univariate analysis and had no impact on the frequency of the metabolic syndrome.

## Conclusion

In summary, this study suggests that the presence of the metabolic syndrome in RA appears to be independent of the use of GCs. It is possible that the disease process itself is the key contributor. This would suggest that effective management of metabolic syndrome in RA is likely to require a two-pronged approach: identification and management of individual classical risk factors, particularly hypertension and hypertriglyceridaemia, as well as aggressive control of inflammatory disease activity with available means, including judicious usage of GCs.

## Abbreviations

anti-TNF: anti-tumour necrosis factor; DAS: disease activity score; DMARD: disease-modifying anti-rheumatic drug; ESR: erythrocyte sedimentation rate; GC: glucocorticoid; HAQ: health assessment questionnaire; HDL: high-density lipoprotein; LE: limited exposure; NCEP III: National Cholesterol Education Programme III; NE: no exposure; OR: odds ratio; RA: rheumatoid arthritis; TG: triglyceride.

## Competing interests

The authors declare that they have no competing interests.

## Authors' contributions

TET participated in the conception and design of the project and in the analysis and interpretation of data and contributed substantially to the drafting of the manuscript. VFP participated in the acquisition, analysis, and interpretation of data. KMJD contributed to the acquisition of data. HRG contributed to the revision of the draft of the manuscript. GDK contributed substantially to the conception and design of the project and to the revision of the draft of the manuscript. All authors read and approved the final manuscript.

## References

[B1] Cobb S, Anderson D, Bauer W (1953). Length of life and cause of death in rheumatoid arthritis. N Engl J Med.

[B2] Kitas GD, Erb N (2003). Tackling ischaemic heart disease in rheumatoid arthritis. Rheumatology (Oxford).

[B3] Van Doornum S, McColl G, Wicks IP (2002). Accelerated atherosclerosis: an extraarticular feature of rheumatoid arthritis?. Arthritis Rheum.

[B4] Stevens RJ, Douglas KM, Saeatzis AN, Kitas GD (2005). Inflammation and atherosclerosis in rheumatoid arthritis. Expert Rev Mol Med.

[B5] (1957). EMPIRE Rheumatism Council: multi-centre controlled trial comparing cortisone acetate and acetyl salicylic acid in the long-term treatment of rheumatoid arthritis; results of three years' treatment. Ann Rheum Dis.

[B6] Kirwan JR (1995). The effect of glucocorticoids on joint destruction in rheumatoid arthritis. The Arthritis and Rheumatism Council Low-Dose Glucocorticoid Study Group. N Engl J Med.

[B7] Bijlsma JW, Van Everdingen AA, Huisman M, De Nijs RN, Jacobs JW (2002). Glucocorticoids in rheumatoid arthritis: effects on erosions and bone. Ann N Y Acad Sci.

[B8] el Shaboury AH, Hayes TM (1973). Hyperlipidaemia in asthmatic patients receiving long-term steroid therapy. Br Med J.

[B9] Panthakalam S, Bhatnagar D, Klimiuk P (2004). The prevalence and management of hyperglycaemia in patients with rheumatoid arthritis on corticosteroid therapy. Scott Med J.

[B10] Sarzi-Puttini P, Atzeni F, Schölmerich J, Cutolo M, Straub RH (2006). Anti-TNF antibody treatment improves glucocorticoid induced insulin-like growth factor 1(IGF1) resistence without influencing myoglobin and IGF1 binding proteins 1 and 3. Ann Rhuem Dis.

[B11] Fraser R, Ingram MC, Anderson NH, Morrison C, Davies E, Connell JM (1999). Cortisol effects on body mass, blood pressure, and cholesterol in the general population. Hypertension.

[B12] Panoulas VF, Douglas KM, Stavropoulos-Kalinoglou A, Metsios GS, Nightingale P, Kita MD, Elisaf MS, Kitas GD (2008). Long-term exposure to medium-dose glucocorticoid therapy associates with hypertension in patients with rheumatoid arthritis. Rheumatology (Oxford).

[B13] Pasquali R, Vicennati V (2008). Steroids and the metabolic syndrome. J Steroid Biochem Mol Biol.

[B14] Rike AH, Mogilishetty G, Alloway RR, Succop P, Roy-Chaudhury P, Cardi M, Kaiser TE, Thomas M, Woodle ES (2008). Cardiovascular risk, cardiovascular events, and metabolic syndrome in renal transplantation: comparison of early steroid withdrawal and chronic steroids. Clin Transplant.

[B15] Varas-Lorenzo C, Rodriguez LA, Maguire A, Castellsague J, Perez-Gutthann S (2007). Use of oral corticosteroids and the risk of acute myocardial infarction. Atherosclerosis.

[B16] Dessein PH, Tobias M, Veller MG (2006). Metabolic syndrome and subclinical atherosclerosis in rheumatoid arthritis. J Rheumatol.

[B17] Reaven GM (1988). Banting lecture 1988. Role of insulin resistance in human disease. Diabetes.

[B18] Reilly MP, Rader DJ (2003). The metabolic syndrome: more than the sum of its parts?. Circulation.

[B19] Sattar N, McConnachie A, Shaper AG, Blauw GJ, Buckley BM, de Craen AJ, Ford I, Forouhi NG, Freeman DJ, Jukema JW, Lennon L, Macfarlane PW, Murphy MB, Packard CJ, Stott DJ, Westendorp RG, Whincup PH, Shepherd J, Wannamethee SG (2008). Can metabolic syndrome usefully predict cardiovascular disease and diabetes? Outcome data from two prospective studies. Lancet.

[B20] Ford ES, Giles WH, Dietz WH (2002). Prevalence of the metabolic syndrome among US adults: findings from the third National Health and Nutrition Examination Survey. JAMA.

[B21] Ilanne-Parikka P, Eriksson JG, Lindström J, Hämäläinen H, Keinänen-Kiukaanniemi S, Laakso M, Louheranta A, Mannelin M, Rastas M, Salminen V, Aunola S, Sundvall J, Valle T, Lahtela J, Uusitupa M, Tuomilehto J, Finnish Diabetes Prevention Study Group (2004). Prevalence of the metabolic syndrome and its components: findings from a Finnish general population sample and the Diabetes Prevention Study cohort. Diabetes Care.

[B22] Meigs JB, Wilson PW, Nathan DM, D'Agostino RB, Williams K, Haffner SM (2003). Prevalence and characteristics of the metabolic syndrome in the San Antonio Heart and Framingham Offspring Studies. Diabetes.

[B23] Prentice AM (2006). The emerging epidemic of obesity in developing countries. Int J Epidemiol.

[B24] Wild S, Roglic G, Green A, Sicree R, King H (2004). Global prevalence of diabetes: estimates for the year 2000 and projections for 2030. Diabetes Care.

[B25] Kakafika AI, Liberopoulos EN, Karagiannis A, Athyros VG, Mikhailidis DP (2006). Dyslipidaemia, hypercoagulability and the metabolic syndrome. Curr Vasc Pharmacol.

[B26] Tracy RP (2003). Inflammation, the metabolic syndrome and cardiovascular risk. Int J Clin Pract Suppl.

[B27] Chung CP, Oeser A, Solus JF, Avalos I, Gebretsadik T, Shintani A, Raggi P, Sokka T, Pincus T, Stein CM (2007). Prevalence of the metabolic syndrome is increased in rheumatoid arthritis and is associated with coronary atherosclerosis. Atherosclerosis.

[B28] Karvounaris SA, Sidiropoulos PI, Papadakis JA, Spanakis EK, Bertsias GK, Kritikos HD, Ganotakis ES, Boumpas DT (2007). Metabolic syndrome is common among middle-to-older aged Mediterranean patients with rheumatoid arthritis and correlates with disease activity: a retrospective, cross-sectional, controlled, study. Ann Rheum Dis.

[B29] Arnett FC, Edworthy SM, Bloch DA, McShane DJ, Fries JF, Cooper NS, Healey LA, Kaplan SR, Liang MH, Luthra HS, Medsger TA, Mitchell DM, Neustadt DH, Pinals RS, Schaller JG, Sharp JT, Wilder RL, Hunder GG (1988). The American Rheumatism Association 1987 revised criteria for the classification of rheumatoid arthritis. Arthritis Rheum.

[B30] Prevoo ML, 't Hof MA, Kuper HH, van Leeuwen MA, Putte LB van de, van Riel PL (1995). Modified disease activity scores that include twenty-eight-joint counts. Development and validation in a prospective longitudinal study of patients with rheumatoid arthritis. Arthritis Rheum.

[B31] Kirwan JR, Reeback JS (1986). Stanford Health Assessment Questionnaire modified to assess disability in British patients with rheumatoid arthritis. Br J Rheumatol.

[B32] (2001). Executive Summary of The Third Report of The National Cholesterol Education Program (NCEP) Expert Panel on Detection, Evaluation, And Treatment of High Blood Cholesterol In Adults (Adult Treatment Panel III). JAMA.

[B33] Stavropoulos-Kalinoglou A, Metsios GS, Koutedakis Y, Nevill AM, Douglas KM, Jamurtas A, Veldhuijzen van Zanten JJ, Labib M, Kitas GD (2007). Redefining overweight and obesity in rheumatoid arthritis patients. Ann Rheum Dis.

[B34] Alberti KG, Zimmet PZ (1998). Definition, diagnosis and classification of diabetes mellitus and its complications. Part 1: diagnosis and classification of diabetes mellitus provisional report of a WHO consultation. Diabet Med.

[B35] Alberti KG, Zimmet P, Shaw J (2005). The metabolic syndrome – a new worldwide definition. Lancet.

[B36] Balkau B, Charles MA (1999). Comment on the provisional report from the WHO consultation. Diabetic medicine.

[B37] Myers J (2003). Cardiology patient pages. Exercise and cardiovascular health. Circulation.

[B38] Rozman B, Praprotnik S, Logar D, Tomsic M, Hojnik M, Kos-Golja M, Accetto R, Dolenc P (2002). Leflunomide and hypertension. Ann Rheum Dis.

[B39] Turesson C, Matteson EL (2006). Is atherosclerosis in rheumatoid arthritis related to disease severity and extraarticular manifestations? Comment on the article by Chung *et al*. Arthritis Rheum.

[B40] Situnayake RD, Kitas G (1997). Dyslipidemia and rheumatoid arthritis. Ann Rheum Dis.

[B41] Georgiadis AN, Papavasiliou EC, Lourida ES, Alamanos Y, Kostara C, Tselepis AD, Drosos AA (2006). Atherogenic lipid profile is a feature characteristic of patients with early rheumatoid arthritis: effect of early treatment – a prospective, controlled study. Arthritis Res Ther.

[B42] Wajchenberg BL, Bosco A, Marone MM, Levin S, Rocha M, Lerario AC, Nery M, Goldman J, Liberman B (1995). Estimation of body fat and lean tissue distribution by dual energy X-ray absorptiometry and abdominal body fat evaluation by computed tomography in Cushing's disease. J Clin Endocrinol Metab.

[B43] Askari A, Vignos PJ, Morkavitz RW (1976). Steroid myopathy in connective tissue disease. Am J Med.

[B44] Afifi AK, Bergman RA, Harvey JC (1968). Steroid myopathy. Clinical, histologic and cytologic observations. Johns Hopkins Med J.

[B45] Rall LC, Roubenoff R (2004). Rheumatoid cachexia: metabolic abnormalities, mechanisms and interventions. Rheumatology (Oxford).

[B46] Roubenoff R, Roubenoff RA, Cannon JG, Kehayias JJ, Zhuang H, Dawson-Hughes B, Dinarello CA, Rosenberg IH (1994). Rheumatoid cachexia: cytokine-driven hypermetabolism accompanying reduced body cell mass in chronic inflammation. J Clin Invest.

[B47] Metsios GS, Stavropoulos-Kalinoglou A, Douglas KM, Koutedakis Y, Nevill AM, Panoulas VF, Kitas GD (2007). Blockade of TNF-alpha in rhuematoid arthritis: the effects on components of rheumatoid cachexia. Rheumatology (Oxford).

[B48] London MG, Muirden KD, Hewitt JV (1963). Serum cholesterol in rheumatic diseases. BMJ.

[B49] Svenson KL, Lithell H, Hallgren R, Selinus I, Vessby B (1987). Serum lipoprotein in active rheumatoid arthritis and other chronic inflammatory arthritides. I. Relativity to inflammatory activity. Arch Intern Med.

[B50] Dursunoglu D, Evrengul H, Polat B, Tanriverdi H, Cobankara V, Kaftan A, Kilic M (2005). Lp(a) lipoprotein and lipids in patients with rheumatoid arthritis: serum levels and relationship to inflammation. Rheumatol Int.

[B51] Rantapaa-Dahlqvist S, Wallberg-Jonsson S, Dahlen G (1991). Lipoprotein (a), lipids, and lipoproteins in patients with rheumatoid arthritis. Ann Rheum Dis.

[B52] Boers M, Nurmohamed MT, Doelman CJ, Lard LR, Verhoeven AC, Voskuyl AE, Huizinga TW, Stadt RJ van de, Dijkmans BA, Linden S van der (2003). Influence of glucocorticoids and disease activity on total and high density lipoprotein cholesterol in patients with rheumatoid arthritis. Ann Rheum Dis.

[B53] Choi HK, Seeger JD (2005). Glucocorticoid use and serum lipid levels in US adults: the Third National Health and Nutrition Examination Survey. Arthritis Rheum.

[B54] Ettinger WH, Hazzard WR (2008). Prednisolone increases very low density lipoproteins and high density lipoproteins in healthy men. Metabolism.

[B55] Bocharov AV, Huang W, Vishniakova TG, Zaitseva EV, Frolova EG, Rampal P, Bertolotti R (1995). Glucocorticoids upregulate high-affinity, high-density lipoprotein binding sites in rat hepatocytes. Metabolism.

[B56] Kantor MA, Cullinane EM, Sady SP, Herbert PN, Thompson PD (1987). Exercise acutely increases high density lipoprotein-cholesterol and lipoprotein lipase activity in trained and untrained men. Metabolism.

[B57] Ettinger WH, Goldberg AP, Applebaum-Bowden D, Hazzard WR (1987). Dyslipoproteinemia in systemic lupus erythematosus. Effect of corticosteroids. Am J Med.

[B58] Stern MP, Kolterman OG, Fries JF, McDevitt  HO, Reaven GM (1973). Adrenocorticol steroid treatment of rheumatic diseases. Effects on lipid metabolism. Arch Intern Med.

[B59] Hench PS, Kendall EC, Slocumb CH, Polley HF (1949). The effect of a hormone of the adrenal cortex (17-hydroxy-11-dehydrocorticosterone; compound E) and of pituitary adrenocorticotropic hormone on rheumatoid arthritis. Proc Staff Meet Mayo Clin.

[B60] Townsend HB, Saag KG (2004). Glucocorticoid use in rheumatoid arthritis: benefits, mechanisms, and risks. Clin Exp Rheumatol.

[B61] Panoulas VF, Metsios GS, Pace AC, John HJ, Treharne GJ, Banks MJ, Kitas GD (2008). Hypertension in rheumatoid arthritis. Rheumatology (Oxford).

[B62] Hafstrom I, Rohani M, Deneberg S, Wornert M, Jogestrand T, Frostegard J (2007). Effects of low-dose prednisolone on endothelial function, atherosclerosis, and traditional risk factors for atherosclerosis in patients with rheumatoid arthritis – a randomized study. J Rheumatol.

[B63] Han C, Robinson DWJ, Hackett MV, Paramore LC, Faeman KH, Bala MV (2006). Cardiovascular disease and risk factors in patients with rheumatoid arthritis, psoriatic arthritis, and ankylosing spondylitis. J Rheumatol.

[B64] Skorodin MS (1993). Pharmacotherapy for asthma and chronic obstructive pulmonary disease. Current thinking, practices, and controversies. Arch Intern Med.

[B65] Svenson KL, Pollare T, Lithell H, Hallgren R (1988). Impaired glucose handling in active rheumatoid arthritis: relationship to peripheral insulin resistance. Metabolism.

[B66] Dessein PH, Joffe BI, Stanwix AE, Christian BF, Veller M (2004). Glucocorticoids and insulin sensitivity in rheumatoid arthritis. J Rheumatol.

[B67] Zarkovic M, Beleslin B, Ciric J, Penezic Z, Stojkovic M, Trbojevic B, Drezgic M, Savic S (2008). Glucocorticoid effect on insulin sensitivity: a time frame. J Endocrinol Invest.

[B68] Svenson KL, Lundqvist G, Wide L, Hallgren R (1987). Impaired glucose handling in active rheumatoid arthritis: effects of corticosteroids and antirheumatic treatment. Metabolism.

